# Current perspectives on recurrent HPV-mediated oropharyngeal cancer

**DOI:** 10.3389/fonc.2022.966899

**Published:** 2022-08-18

**Authors:** Theresa Guo, Stephen Y. Kang, Ezra E. W. Cohen

**Affiliations:** ^1^ Department of Otolaryngology-Head and Neck Cancer, Moores Cancer Center, University of California, San Diego, San Diego, CA, United States; ^2^ Department of Otolaryngology-Head and Neck Surgery, Ohio State University Wexner Medical Center, Ohio State University, Columbus, OH, United States; ^3^ Division of Medical Oncology, Moores Cancer Center, University of California, San Diego, San Diego, CA, United States

**Keywords:** oropharyngeal cancer (OPSCC), recurrent disease, surgical salvage, immunotherapy, human papillomavirus (HPV), head neck squamous carcinoma

## Abstract

In the recent years, the prevalence of HPV-positive oropharyngeal squamous cell carcinoma (OPSCC) has increased significantly. Currently, nearly 80-90% of all oropharynx tumors are HPV-positive. In addition, it is now recognized that HPV-positive tumor status is associated with good prognosis and improved response to chemoradiation. However, within this setting, there are still patients with HPV-positive OPSCC who will experience recurrence. With the increasing incidence of HPV-mediated OPSCC, recurrent HPV disease is also becoming more prevalent and there is an increasing need to understand the unique presentation and treatment of recurrent HPV-mediated disease. In this review, we will discuss epidemiology of recurrent HPV-positive OPSCC, role of surgical salvage, re-irradiation, and the role of upcoming novel treatments and immunotherapy. Historically, recurrent oropharyngeal disease has been associated with poor prognosis and high morbidity. However, recent advances have transformed the landscape for salvage treatment of HPV-mediated OPSCC. Liquid biomarkers offer potential for early detection of recurrence, robotic techniques may reduce morbidity of surgical salvage, improvements in re-irradiation approaches reduce toxicities, and novel immune based therapies on the horizon are offering promising results. These advances combined with the improved prognosis of HPV-positive disease offer to transform our approach to recurrent disease of the oropharynx.

## Introduction

Despite the improved prognosis conferred by human papillomavirus (HPV)-positive tumor status, recurrences and distant failures still occur in this population with progression free survival of 72-74% at 3-years (1, 2). Recurrence rates in HPV-positive oropharyngeal squamous cell carcinoma (OPSCC) patients are about half that of HPV-negative patients, with respect to both locoregional and distant failure. Results from RTOG 0522 showed that in HPV-positive compared to HPV-negative patients, 3-year locoregional failure rates were 17.3% vs 32.5% (P <.001) and distant metastatic rates were 6.5% vs. 17.0% (p=.005) (2). Recurrences tend to occur later in HPV-positive patients (3), but regardless of HPV-tumor status a majority of recurrences occur within 2 years of primary treatment (3–5).

Patterns of recurrence with regard to local, regional and distant sites do not differ significantly by HPV status (3, 4, 6). With regard to distant progression, the lung is the most common site of distant metastasis for both HPV-positive and HPV-negative patients (3–5, 7). Some studies have described unusual patterns of distant metastatic disease in HPV-positive OPSCC, including unusual sites such as brain metastases (8), and disseminated metastases to multiple organs sites (9, 10). However, recent evidence has not confirmed unusual metastatic disease patterns specific to the HPV-positive population (3–5, 7). Distant recurrences do occur later in HPV-positive OPSCC (3, 5), though the longer overall survival of HPV-positive patients may contribute to development these late recurrences (6, 11).

Smoking status and greater disease burden at the time of primary treatment are the main risk factors for recurrence in HPV-positive disease (1). Ang et al. recognized early on the impact of smoking history on HPV-positive disease, defining an intermediate risk group to include HPV-positive patients with >10 pack year smoking history (1). Subsequent studies have confirmed the increased risk of recurrence for smokers in this population (12). In prior studies, AJCC 7^th^ edition stage was not independently associated with progression free survival in either p16-positive and p16-negative OPSCC patients (13). However, larger primary tumor burden, especially T4 disease, has been associated with increased risk for recurrence (14–16). High-risk nodal features have also been associated with risk of recurrence and distant progression including presence of N3 disease, extra nodal extension (ENE), and retropharyngeal adenopathy (16–18).

## Detection of recurrent disease

Recommended schedule of surveillance for oropharyngeal cancer does not currently differ by HPV status (19). One study demonstrated that recurrent HPV-positive disease was mainly diagnosed by imaging compared to HPV-negative disease which was mainly diagnosed through physical examination (3). As discussed above, patterns of recurrence generally do not differ between HPV-positive and HPV-negative disease, with majority of recurrences occurring within 2 years. However, recurrences may occur later in HPV-positive patients, and there is some evidence for late distant metastasis. Given these findings, some advocate for extended surveillance of HPV-positive patients.

Recently there has been increasing interest in the use of HPV DNA (primarily HPV-16) as a biomarker of treatment response and recurrent disease. Early studies established the relationship between detectable serum or saliva HPV DNA with increased risk for recurrent disease (20). In a prospective study of 396 patients, oral rinses were able to detect HPV DNA in 80% of HPV-positive patients at diagnosis, and persistent HPV detection after treatment was significantly associated with decreased recurrence free and overall survival (21). HPV protein (E6 & E7) antibody levels have also been proposed as a biomarker for monitoring disease. These antibody levels have been shown to decrease in both serum and saliva after treatment (22, 23). The biomarker with that has now been most studied is HPV cell free or circulating tumor (ct) DNA. Improvements in PCR techniques including digital droplet PCR and next generation sequencing have facilitated high sensitivity of these assays (24). For HPV-positive patients, HPV ctDNA can be detected at diagnosis in about 65-90% of patients (24). While patients with higher TNM stage are more likely to have detectable HPV ctDNA at baseline (25, 26), ctDNA can also be detected in early stage disease (27). In these patients, there is a rapid decline in ctDNA after both surgical treatment (28) and primary chemoradiation (29). Those with persistent HPV ctDNA after treatment were more likely to have adverse pathologic features, and increased risk for recurrence (28, 29). A recent study of 1076 patients evaluated circulating HPV-DNA serially after definitive therapy. In patients who were otherwise without evidence of disease, of those with positive HPV ctDNA, 93% were identified to have occult recurrence (30). ctDNA has also been proposed as an adjunct to post-treatment imaging for evaluation of treatment response (31). The utility of ctDNA for detection of residual or recurrent disease has also been demonstrated in HPV associated cervical cancer (32) and anal squamous cell carcinoma (33). While additional clinical validation is needed prior to incorporation to clinical practice to augment surveillance of HPV-positive disease, early results are promising (**Table 1**). Additional studies are also needed to better understand patients who do not have detectable HPV ctDNA at baseline, the role of ctDNA levels at the time of diagnosis, the role of ctDNA for early diagnosis and screening, and how to integrate these tests into current surveillance practices.

**Table 1:** HPV ctDNA clearance and recurrence detection in OPSCC.

Study	n	Main findings
Damerla et al, 2019 (27)	97	HPV ctDNA was detectable in 93% of patients, including those with low volume disease (T1-2, or single node disease). ctDNA was rapidly cleared from majority of patients by week 7 of CRT
Chera et al, 2019 (29)	103	ctDNA evaluated in patients who received primary chemoradiation. All patients with favorable clearance (>200 copies/mL at baseline) and >95% clearance had no persistent or recurrent disease. Those without rapid clearance had adverse clinical risk factors (T4, >10 pack years) and higher rate of persistent or recurrent disease.
O’Boyle et al, 2022 (28)	33	Kinetics were evaluated in patients who underwent primary surgery for HPV+ OPSCC demonstrating rapid decrease of ctDNA by post operative day (POD) 1. Those with elevated ctDNA on POD1 were more likely to have high risk pathologic features
Berger et al, 2022 (30)	1076	Multi-institutional retrospective assessment of cell-free tumor tissue modified (TTMV)-HPV DNA following definitive therapy. 80 patients had positive test upon surveillance. Of these, 21 had clinically detected recurrence. Of remaining 59 patients, 93% were subsequently confirmed to have recurrence.

## Considerations for management of recurrent disease

After recurrence, HPV-positive patients still demonstrate improved outcomes compared to their HPV-negative counterparts (3–5, 7, 42, 56). Other factors that contribute to improved overall survival include longer disease free interval and lower disease burden at time of recurrence (3, 57, 58). Treatment of recurrent disease with surgical salvage with or without adjuvant therapy when feasible, is associated with significant improvement in survival after both locoregional and distant recurrence (3, 4, 7, 57, 59, 60). The retrospective nature of salvage surgery studies should be recognized, where patients with localized disease burden and higher performance status are more likely to be selected for surgical salvage. In addition, the recent FDA approval of immunotherapy for treatment of recurrent metastatic head and neck cancer may change the landscape of systemic treatment options in the future. A summary of salvage treatment options is detailed in **Table 2**.

**Table 2:** Salvage treatment options.

Surgical Salvage	
Locoregional disease	Associated with improved survival in retrospective studies [2yr OS 78.9%] (3); TORS assisted surgery and free flap reconstruction may improve functional outcomes (34)
Distant metastasis	Can be considered for solitary metastasis, with associated improvement in survival [2yr OS 86.5%] (3, 7)
**Radiation**	
Locoregional disease	Re-irradiation with IMRT (35, 36), proton (37, 38) and SBRT (39, 40) provide promising locoregional control and tolerable toxicity [2 yr LRPFS 30.9-52%; 2yr OS 54.6-69%]; Advantage of surgical resection prior to re-irradiation are not clearly established
Distant metastasis	SBRT can be considered for solitary or oligometastatic disease with up to 75% response rate (41) and over 50% 2-yr OS in those without LR recurrence (35);
**Systemic therapy: For patients with unresectable disease, without re-irradiation options or distant metastasis**	
Cytotoxic chemotherapy	EXTREME regimen (cetuximab, platinum, 5-FU) previous standard of care (42)
Immune checkpoint inhibition (anti-PD1/PDL1)	Pembrolizumab or Nivolumab, now approved for first line treatment of recurrent/metastatic HNSCC (43, 44). Improved response in PD-L1 expressing tumors (45–47), HPV not clearly associated with response (48, 49). Some patients may have sustained responses May be combined with chemotherapy (platinum & 5-FU) (50) May be combined with cetuximab based on antibody dependent cellular toxicity (51)
Novel immune based therapies under investigation	Therapeutic HPV vaccines targeting E6/E7 antigens may be combined to boost response to anti-PD1 therapies (52) Engineered T-cells expressing HPV-16 E7 TCR (53) Other immune based targets to be combined with anti-PD1 therapy: TIGIT, CD47, LAG3, KIR (54, 55)

### Surgical salvage

In cases of recurrent OPSCC, surgical salvage with curative intent should be offered when feasible. Historically, recurrent oropharyngeal disease has been associated with poor prognosis with lower rates of survival and higher rates of surgical complication compared to other head and neck subsites (57, 61, 62). In this context, surgical salvage was often considered unacceptably morbid for minimal benefit. In one case series of patients treated with surgical salvage for OPSCC did show improved survival, however, 46% experienced post-operative complications and 67% of patients developed another recurrence at a median of 8 months (57). However, the improved prognosis of increasingly prevalent HPV-positive disease has significantly improved overall survival for recurrent OPSCC. One meta-analysis demonstrated an increase in 5-year overall survival for recurrent OPSCC from 18% to 51% in patients treated before and after 2000 (63). Concurrently, advancements in minimally invasive transoral robotic techniques have reduced surgical morbidity through minimally invasive approaches (64, 65). With these changes in the modern era, surgical salvage has fallen back into favor. Multiple retrospective studies have demonstrated the significant survival benefit associated surgical salvage with 5-year survival rates of 43-49% compared to 16% for non-surgical therapy (62, 66, 67). Ability to achieve negative margins is one of the most important predictor of surgical salvage success (59, 67–69).

TORS for recurrent oropharyngeal disease has been associated with decreased post-operative complications, including lower long-term tracheostomy and feeding tube dependence (34, 70). White et al. performed a multi-institutional matched analysis comparing TORS-assisted salvage surgery compared to open surgery and found that TORS-assisted surgery reduced tracheostomy use, feeding tube use, and reduced hospital length of stay. In addition, TORS-assisted surgery in this study was also associated with improved oncologic outcomes with decreased positive margins and improved recurrence free survival (34). Multiple case series have demonstrated feasibility of TORS assisted surgical salvage in the oropharynx, which may include free flap reconstruction with TORS assisted flap inset (64, 65, 70).

A patient’s burden of recurrent disease and functional status play an important role in selecting patients most likely to benefit from surgical salvage. As previously discussed, selection for surgical salvage should foremost consider ability to achieve negative margins (57–60, 67–69). Other markers of aggressive tumor behavior such as short disease free interval or persistence, lymphovascular invasion and positive cervical nodal recurrence portend worse survival following salvage (57, 59, 71, 72). In addition, older patient age and laryngopharyngeal dysfunction are significant negative predictors of survival in the salvage setting (57, 72). Heft Neal et al. proposed a classification system which predicted survival following salvage surgery for recurrent oropharyngeal cancer following radiation. Class I patients (disease free interval > 2 years) had the highest five-year overall survival at 47% compared with 0% of Class III patients with short disease free interval of <2 years and laryngopharyngeal dysfunction (72). Other studies have also demonstrated that G-tube dependence is associated with decreased overall survival after surgical salvage (71, 73).

Given that patients with advanced primary disease are more likely to recur, 90-95% of patients who recur will have received prior radiation either in the primary or adjuvant setting (3, 59). In this setting, regional or free flap reconstruction of surgical defects are recommended for reconstruction to reduce risk of fistula and prevent vessel exposure (62, 64, 74). Free tissue transfer significantly reduces morbidity and major complications in salvage laryngeal surgery (75, 76) and has similarly been employed in salvage oropharyngeal surgery to bring vascularized tissue to the irradiated wound bed with high success rates (62). One of the most common reconstructive strategies involves an L-shaped soft tissue template as described by Chepeha et al. (77) Common donor sites include the radial forearm, anterolateral thigh, and lateral arm. The authors describe the three fundamental goals of primary oropharyngeal reconstruction: obliteration of the oropharynx, preservation of nasopharyngeal competence, and maintenance of base of tongue mobility (77). In the surgical salvage oropharyngectomy, where greater wound contraction is encountered, free tissue transfer plays a vital role in achieving these reconstructive principles. TORS techniques can assist with free flap inset, with vessel anastomosis performed in an open neck (78). In addition to free tissue transfer, use of the submental island flap has also been described after TORS (79). These techniques require specialized expertise and equipment; however, with appropriate patient selection they can reduce surgical morbidity (34, 70). These minimally invasive approaches are not always feasible, and patients with more extensive disease, severe trismus or requiring bony resection will require open resection. In the setting of prior radiation and when free flap reconstruction is required, tracheostomy and feeding tube placement are routinely utilized in conjunction with salvage surgery (57, 62, 70). Most patients can achieve decannulation and oral diet by six-months after surgery, but these rates are lower in those undergoing open surgery and in those with greater disease burden (57, 71).

Isolated neck recurrence may provide greater chance at complete resection of recurrent disease, however nodal recurrence is associated with high rates of further recurrence and metastasis (61, 73). Prior studies including all head and neck subsites have demonstrated that salvage neck dissection was associated with improved survival compared to non-surgical treatment (80). However, risk of recurrence after salvage neck dissection was greater when the neck was previously dissected (81).

For patients with resectable recurrence after prior radiation treatment, there is strong evidence that adjuvant therapy following salvage surgery reduces progression free survival and is recommended by NCCN guidelines (19, 82). OPSCC patients who experience recurrence are at high risk for developing second recurrence (68), and use of postoperative radiation is associated with improved survival after surgical salvage (72). Reconstruction in salvage surgery for recurrent local or neck disease can assist in reducing morbidity of re-irradiation in the adjuvant setting by providing vascularized tissue coverage (83).

### Re-irradiation

For patients with unresectable disease or who are unable to undergo surgical resection, re-irradiation with or without chemotherapy is an option that has historically demonstrated limited benefit for non-nasopharyngeal sites, and carries high rates of toxicity (35, 61). Trials combining chemotherapy with hyper-fractionated reirradiation for recurrent head and neck cancer had fairly low overall survival (15.2% at two years), although patients with longer disease free interval had better outcomes (84). Advancements in radiation therapy technology have improved outcomes. Patients receiving intensity modulated radiation therapy (IMRT) in the re-irradiation setting demonstrate improved locoregional control (52% vs 20% at 2 years), and patients who underwent gross total resection also trended towards improved locoregional control (35). While salvage surgery has been associated with improved progression free survival (36), the advantages of surgical resection prior to re-irradiation have not been universally reported when compared to re-irradiation with curative intent (85). Re-irradiation with proton therapy has also recently demonstrated promising locoregional control results (68% locoregional control at 1 year) with tolerable toxicity profiles (37, 38). Additionally, stereotactic body radiotherapy (SBRT) has also demonstrated comparable disease control in the recurrent setting, with improved outcomes in HPV-positive patients (39, 40).

### Distant metastasis

When distant metastasis occurs, generally systemic treatment options are favored. However, one recent study demonstrated increased survival associated with surgical salvage for distant metastases from OPSCC, with an increase of median survival from 12.5 to 35 months (3). A majority (87%) of patients were HPV-positive, and surgery included lung nodule resection, mediastinal lymphadenectomy, hepatectomy and craniotomy. Another review of OPSCC patients with distant metastases demonstrated significantly improved 3-year disease specific survival (40% vs 8%) in patients receiving curative therapy (surgery with negative margins or definitive radiation) for distant disease compared to palliative systemic treatment (7). For patients with oligometastatic lung disease, SBRT has shown up to 75% response rates (41) and over 50% 2-year overall survival in those without locoregional recurrence (35). While these retrospective studies are inevitably subject to selection bias, they still support the potential survival benefit that surgery and definitive radiation can offer select OPSCC patients with limited distant metastases.

### Systemic therapy

For patients who do not have surgical or re-irradiation options, systemic chemotherapy regimens are the mainstay for recurrent metastatic head and neck cancer. The EXTREME regimen including cetuximab, platinum and 5-fluorouracil (5-FU) had been the standard treatment regimen for the past decade (42, 86). The EXTREME regimen demonstrated median overall survival of 12.6 months for p16+ patients and 9.7 months for p16- patients; both groups benefited from the addition of cetuximab to platinum/5-FU (42). Recently, the advent of immunotherapy through anti-PD1/PDL1 checkpoint inhibition has provided promising new options. For patients refractory to platinum therapy or cetuximab, overall response rates were 13-18% and overall survival was significantly improved for patients receiving immunotherapy (pembrolizumab or nivolimab) compared to standard chemotherapy (43, 44). Subgroup analysis demonstrated higher response rates in patients whose tumors expressed PD-L1, irrespective if this was on cancer or stromal cells (45–47). Despite, having on average a higher rate of immune cell infiltrate, the impact of HPV tumor status on immunotherapy response rate has not been clearly established, with some studies showing higher response rates in HPV-positive patients (48) and others showing greater survival benefit for HPV-negative patients (49). With the establishment of anti-PD1 agents in platinum refractory disease, pembrolizumab was tested either as monotherapy or in combination with chemotherapy (platinum and 5-FU) in patients with recurrent disease who were treatment naïve (87). Compared to the EXTREME regimen, pembrolizumab monotherapy improved overall survival in patients whose tumors expressed PDL1 and pembrolizumab with chemotherapy improved overall survival in all patients, irrespective of tumor PDL1 expression. Nonetheless, higher tumor PDL1 expression was associated with a greater benefit in both pembrolizumab-containing treatment arms further establishing this a predictive biomarker. It is also notable that, in long-term follow up, approximately 25% of subjects treated with pembrolizumab on either arm were alive suggesting that there is long-term benefit to immunotherapy (50).

In an effort to integrate the potential benefit of T cell stimulation *via* PD1 blockade and antibody dependent cellular cytotoxicity of IgG1 antibodies, Sacco et al. combined pembrolizumab and cetuximab in recurrent/metastatic HNSCC patients who were refractory to or poor candidates for platinum (51). This doublet regimen proved to be remarkably active with an objective response rate of 45% and median overall survival of 18 months, regardless of HPV status. The activity of PD1/EGFR inhibition has been confirmed by two independent subsequent studies and now represents a treatment option for patients who are not good candidates for platinum-based therapy in the recurrent/metastatic setting (88, 89).

Specific therapies for HPV related HNSCC are also being developed taking advantage of the unique antigens of a virally induced malignancy. These include therapeutic HPV vaccines and engineered T cells. Several therapeutic HPV vaccines are being tested in clinical trials with early promising results. For instance, ISA101b is a synthetic long peptide vaccine targeting HPV-16 E6 and E7 antigens which demonstrated an objective response rate in HPV-related oropharynx cancer of 33% in combination with nivolumab (52). This vaccine is currently being evaluated in a randomized study of cemiplimab, an anti-PD1 antibody, with or without ISA101b.

Cloning the T cell receptor (TCR) for a given HPV antigen and HLA type has introduced the possibility of engineering T cells to express the relevant TCR. These strategies have been tested in clinical trials using HPV-16 E6 and E7 TCRs. Interestingly, a clinical trial of an HPV-16 E7 TCR recently demonstrated a 50% response rate in refractory tumors (53). While this activity is promising, analysis of tumor biopsies at progression revealed emergence of resistance through loss of HPV-16 antigen presentation; a consequence that TCR strategies will need to overcome to demonstrate long-term benefit.

In this evolving field, novel combinations are under study that attempt to leverage the benefits demonstrated with anti-PD1 blockade including adding agents that target TIGIT, CD47, and LAG3 (54). Furthermore, applications for immunotherapy will continue to transform treatment options for patients with recurrent disease such as considerations for neoadjuvant immunotherapy before salvage surgery, or maintenance immunotherapy after salvage treatment. Promising results have been obtained administering neoadjuvant and adjuvant nivolumab and lirilumab, an anti-KIR antibody, in patients with recurrent and resectable HNSCC with 1-year disease-free and overall survival of 55% and 85%, respectively (55). These will need to validated in randomized studies but provides encouragement for improved outcomes in these difficult to treat patients.

## Conclusions

Although HPV-mediated OPSCC is associated with improved prognosis and decreased rates of recurrence, recurrent disease still occurs. With the increasing incidence of HPV-mediated OPSCC, recurrent HPV disease is also becoming more prevalent and there is an increasing need to understand the unique presentation and treatment of recurrent HPV disease. Recurrences in HPV-positive patients may occur later, and patients may have improved outcomes after recurrence compared to HPV-negative counterparts. Emerging data demonstrates that detection of recurrences may be aided by evaluation of circulating tumor HPV DNA. Surgical salvage is the preferred treatment when feasible, and robotic approaches can decrease morbidity. New advances in re-irradiation and immune based therapies are offering promising results for this patient population.

## Data availability statement

The original contributions presented in the study are included in the article/supplementary material. Further inquiries can be directed to the corresponding author.

## Author contributions

All authors (TG, SK, and EC) provided substantial contributions to the conception of this work, drafting and writing as well as approval of publication.

## Funding

TG is supported by 1KL2TR001444 from NIH.

## Conflict of interest

The authors declare that the research was conducted in the absence of any commercial or financial relationships that could be construed as a potential conflict of interest.

## Publisher’s note

All claims expressed in this article are solely those of the authors and do not necessarily represent those of their affiliated organizations, or those of the publisher, the editors and the reviewers. Any product that may be evaluated in this article, or claim that may be made by its manufacturer, is not guaranteed or endorsed by the publisher.
